# Mueller-matrix-based polarization imaging and quantitative assessment of optically anisotropic polycrystalline networks

**DOI:** 10.1371/journal.pone.0214494

**Published:** 2019-05-16

**Authors:** Mariia Borovkova, Larysa Trifonyuk, Volodymyr Ushenko, Olexander Dubolazov, Oleg Vanchulyak, George Bodnar, Yurii Ushenko, Olena Olar, Olexander Ushenko, Michael Sakhnovskiy, Alexander Bykov, Igor Meglinski

**Affiliations:** 1 Optoelectronics and Measurement Techniques, Faculty of Information Technology and Electrical Engineering, University of Oulu, Oulu, Finland; 2 Rivne State Medical Center, Rivne, Ukraine; 3 Department of Correlation Optics, Chernivtsi National University, Chernivtsi, Ukraine; 4 Department of Optics and Publishing Business Chernivtsi National University, Chernivtsi, Ukraine; 5 Department of Forensic Medicine, Bukovinian State Medical University, Chernivtsi, Ukraine; 6 Department of Computer Science, Chernivtsi National University, Chernivtsi, Ukraine; 7 National Research Tomsk State University, Interdisciplinary Laboratory of Biophotonics, Tomsk, Russia; 8 Institute of Engineering Physics for Biomedicine (PhysBio), National Research Nuclear University “MEPhI”, Moscow, Russia; Pennsylvania State Hershey College of Medicine, UNITED STATES

## Abstract

We introduce a Mueller-matrix imaging polarization-based approach for the quantitative digital screening of the polycrystalline structure of fibrillary-based biological tissues *in vitro*. The morphometric evaluation of histological sections of myocardium was performed utilizing the high-order statistical moments calculated based on the spatial distribution of linear and circular birefringence and dichroism obtained experimentally. We demonstrate that spatial distributions of phase of light and optical anisotropy of scattering inherent to fibrillar networks of myocardium at different necrotic stages can be effectively used as a quantitative marker of stages of myosin fibril degradation. Processing the images of phase of light scattered in biological tissues with high order statistical analysis provides a functional tool for the quantitative characterization of necrotic conditions of the myocardium.

## Introduction

In addition to the conventional methods, typically used for quantitative characterization of turbid tissue-like phase-inhomogeneous scattering media, the new promising approaches, such as optical polarimetry and Mueller-matrix imaging are used extensively for screening of optically anisotropic biological samples [1–7]. Sufficient progress was achieved in the theoretical description and modeling of the optical radiation propagation in a turbid tissue-like scattering medium [8–11], as well as in polarimetric microscopy studies of protein fibrils of optically thin (non-depolarizing) tissue slices [12]. The application of the circularly polarized laser light is extended meaningfully for the functional histological examination of paraffin-embedded tissue blocks [13,14]. An important feature of the unified quantitative assessment of morphological images of biological tissues containing collagen fibrils is the quantitative assessment of fibrils’ size, density [15], and spatial orientation [16]. No less relevant direction in the use of Muller-matrix polarimetry is the study of the effectiveness of the diagnosis of human colon cancer [17]. The analysis of Mueller-matrix images of histological sections of biological tissues was continued by means of high-order statistical analysis of tissue samples with optical anisotropy [18–20]. Based on the acknowledged peculiarities of polarized light propagated in turbid tissue-like scattering medium [13,14], the new diagnostic modality for the cancer screening and characterization of abnormalities in biological tissues due to cancer aggressiveness has been suggested [21–27]. This approach is based on the analysis and differentiation of fully polarized and completely depolarized Mueller-matrix components [28] and can be potentially utilized for the reconstruction and quantitative evaluation of the polycrystalline structure of fibrillar network. Current study is dedicated to the development of the Mueller-matrix imaging approach and the reconstruction of the spatial distribution of optical anisotropy associated with the necrotic variations in histological sections of the fibrillary-based tissue samples, such as myocardium.

Despite the fact that nowadays microscopy-based histochemical screening of postmortem tissues is the leading technique in morphological and forensic diagnostic studies [29], this approach does not exceed 65%-70% of accuracy and is rather time-consuming [30].

## Method and materials

### Basic equations and theoretical remarks

Typically, optical anisotropy of biological tissues is defined as linear and circular birefringence (phase anisotropy) and linear and circular dichroism (amplitude anisotropy) [1–7, 15–20]. Circular birefringence and dichroism appear due to the spiral-like structure of protein molecules, whereas linear birefringence and dichroism are associated with the spatially ordered fibrillar networks in biological tissues.

The theoretical basis of the Mueller-matrix imaging approach to describe the interaction of optical radiation with depolarizing layers is widely presented in literature [31–39]. Specifically, in case of multiple scattering, the Mueller matrix of a depolarizing layer varies along the direction of light propagation. This can be described analytically, as:

$$\frac{d\left\{M\right\}\left(z\right)}{dz}=\left\{M\right\}\left(z\right)\left\{m\right\}\left(z\right),$$ (1)

Where {*M*}(*z*) is the Mueller matrix of the object in the plane *z*(0≤*z*≤*l*); {*m*}(*z*) is differential Mueller matrix.

The ratio between the matrices {*M*}(*z*) and {*m*}(*z*) is given by:

$$\left\{M\right\}\left(z\right)=\exp\left(\left\{m\right\}\left(z\right)\right)=\exp\left(\langle \left\{m\right\}\rangle +\langle \left\{\Delta m^{2}\right\}\rangle \right).$$ (2)

Here, 〈{*m*}〉 is the polarization part, and 〈{Δ*m*^2^}〉 is the depolarization part of the Mueller matrix {*M*}(*z*) of the scattering layer.

The differential matrix {*m*}(*z*) consists of six basic polarization properties that fully describe optical anisotropy of the biological layer:

$$\begin{array}{l} \langle \left\{m\right\}\rangle =\left\|\begin{array}{cccc} m_{11}; & m_{12}; & m_{13}; & m_{14};\\ m_{21}; & m_{22}; & m_{23}; & m_{24};\\ m_{31}; & m_{32}; & m_{33}; & m_{34};\\ m_{41}; & m_{42}; & m_{43}; & m_{44} \end{array}\right\|=\\ =\left\|\begin{array}{cccc} {\left(0\right)}_{11}; & {\left(LD_{0;90}\right)}_{12}; & {\left(LD_{45;135}\right)}_{13}; & {\left(CD_{⊗;⊕}\right)}_{14};\\ {\left(LD_{0;90}\right)}_{21}; & {\left(0\right)}_{22}; & {\left(CB_{⊗;⊕}\right)}_{23}; & {\left(-LB_{45;135}\right)}_{24};\\ {\left(LD_{45;135}\right)}_{31}; & {\left(-CB_{⊗;⊕}\right)}_{32}; & {\left(0\right)}_{33}; & {\left(LB_{0;90}\right)}_{34};\\ {\left(CD_{⊗;⊕}\right)}_{41}; & {\left(LB_{45;135}\right)}_{42}; & {\left(-LB_{0;90}\right)}_{43}; & {\left(0\right)}_{44} \end{array}\right\| \end{array}.$$ (3)

Here, *LD*_0,90_, *LD*_45,135_ and *LB*_0,90_, *LB*_45,135_ stand for the linear dichroism and birefringence; *CD*_⊗;⊕_ and *CB*_⊗;⊕_ are the circular dichroism and birefringence of the optically anisotropic component of the biological layer for the linearly (0^0^÷90^0^; 45^0^÷135^0^), circularly right- (⊗) and left- (⊕) polarized unit vectors.

The analysis of Eqs (1)–(3) is resulted in an expression in form of logarithmic matrix:

$$\begin{array}{l} \ln\left\{M\left(z\right)\right\}=\langle \left\{m\right\}\rangle z+0.5\langle \left\{\Delta m^{2}\right\}\rangle z^{2}=\\ =0.5\left(\left(\ln\left\{M\left(z\right)\right\}-G\ln{\left\{M\left(z\right)\right\}}^{T}G\right)+\left(\ln\left\{M\left(z\right)\right\}+G\ln{\left\{M\left(z\right)\right\}}^{T}G\right)\right). \end{array},$$ (4)

Here, *G* = *diag*(1,−1,−1,−1). is the metric of Minkowsky matrix [39], *T* is the transpose operation.

Taking into account Eqs (2), (4), the polarization component 〈{*m*}〉 of the logarithmic matrix algorithm ln{*M*(*z*)} is described:

$$\langle \left\{m\right\}\rangle =l^{-1}\left\|\begin{array}{cccc} {\left(0\right)}_{11}; & {\left(\ln\left(M_{12}M_{21}\right)\right)}_{12}; & {\left(\ln\left(M_{13}M_{31}\right)\right)}_{13}; & {\left(\ln\left(M_{14}M_{41}\right)\right)}_{14};\\ {\left(\ln\left(M_{12}M_{21}\right)\right)}_{21}; & {\left(0\right)}_{22}; & {\left(\ln\left(\frac{M_{23}}{M_{32}}\right)\right)}_{23}; & {\left(\ln\left(\frac{M_{24}}{M_{42}}\right)\right)}_{24};\\ {\left(\ln\left(M_{13}M_{31}\right)\right)}_{31}; & {\left(\ln\left(\frac{M_{32}}{M_{23}}\right)\right)}_{32}; & {\left(0\right)}_{33}; & {\left(\ln\left(\frac{M_{34}}{M_{43}}\right)\right)}_{34};\\ {\left(\ln\left(M_{14}M_{41}\right)\right)}_{41}; & {\left(\ln\left(\frac{M_{42}}{M_{24}}\right)\right)}_{42}; & {\left(\ln\left(\frac{M_{43}}{M_{34}}\right)\right)}_{43}; & {\left(0\right)}_{44} \end{array}\right\|.$$ (5)

From Eqs (3) and (5) we obtain a way for the polarization reconstruction of the phase (Δ*n*_0;90_; Δ*n*_45;135_; Δ*n*_⊗;⊕_) and amplitude (Δ*μ*_0;90_; Δ*μ*_45;135_; Δ*μ*_⊗;⊕_) anisotropy parameters:

$$\Phi \equiv \left\{ \begin{array}{l} \Delta n_{0,90}=\frac{\lambda }{2\pi l}\ln\left(\frac{M_{34}}{M_{43}}\right);\\ \Delta n_{45;135}=\frac{\lambda }{2\pi l}\ln\left(\frac{M_{24}}{M_{42}}\right);\\ \Delta n_{⊗;⊕}=\frac{\lambda }{2\pi l}\ln\left(\frac{M_{23}}{M_{32}}\right); \end{array}\right.$$ (6)

$$A\equiv \left\{ \begin{array}{l} \Delta \mu_{0,90}=\frac{\lambda }{2\pi l}\ln\left(M_{12}M_{21}\right);\\ \Delta \mu_{45;135}=\frac{\lambda }{2\pi l}\ln\left(M_{13}M_{31}\right);\\ \Delta \mu_{⊗;⊕}=\frac{\lambda }{2\pi l}\ln\left(M_{14}M_{41}\right). \end{array}\right.$$ (7)

The spatial distributions Φ(*m*×*n*) and A(*m*×*n*), obtained within a set of pixels (*m*×*n*) of the photosensitive area of a digital camera, are further referred as the polarization-phase images of polycrystalline structure (optically anisotropic medium with a probability distribution of the directions of the optical axes and phase shifts) of fibrillar networks of biological tissues.

### Experimental method

**Fig 1:**
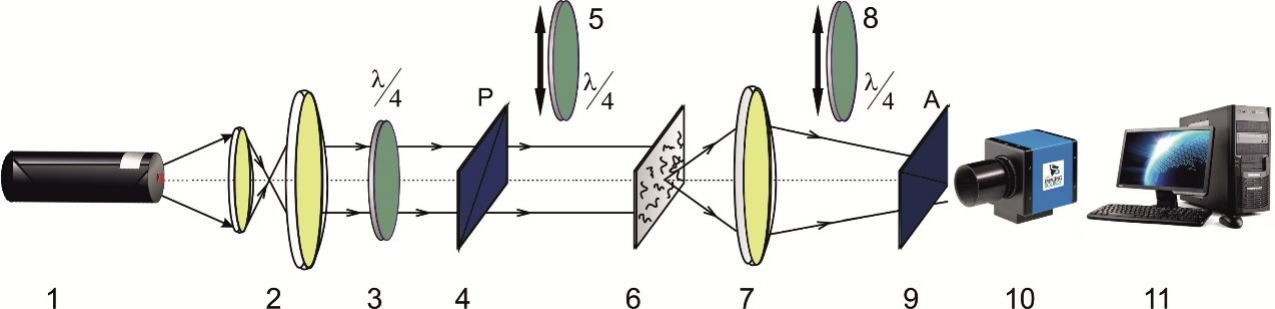
Schematic presentation of the Mueller-polarimeter experimental setup, including “blue” laser diode (1), collimator (2), stationary quarter-wave plate (3), mechanically movable quarter-wave plates (5) and (8), polarizer (4) and analyzer (9), object of investigation (6), infinity corrected strain-free objective with tube lens (7), CCD camera (10), personal computer (11).

Experimental studies were performed using the classic polarimetry setup [18,19] schematically presented in Fig 1.

The tissue sample (6) was illuminated by the laser beam (diameter ∅ = 10^4^ μm) generated by the “blue” laser diode (1) at the wavelength *λ* = 0.405*μm* with power *W* = 50*μW* (see Fig 1). The polarized light source consisted of quarter-wave plates (Achromatic True Zero-Order Wave-plates: (3), (5), (8)) and the polarizer (4). Histological slices of biological tissues (6) were placed in the focal plane of the strain-free objective Nikon CFI Achromat P (7), with the working distance of 30mm, *NA* of 0.1, and 4x magnification). The strain-free objective formed the spatial intensity distribution at the plane of the light-sensitive CCD-camera (Imaging Source DMK 41AU02.AS, monochrome 1/2" CCD, Sony ICX205AL (progressive scan); resolution of *m*×*n* = 1280x960; light-sensitive plate size of 7600x6200 μm; sensitivity of 0.05 lx; dynamic range of 8 bit; SNR of 9 bit, deviation of photosensitive characteristics from the linear ones did not exceed 5%). The camera provided the images of histological slices of biological tissues for geometrical sizes of 2–2000 μm.

Polarization filtration of the images of the histological slices of biological tissues was carried out utilizing the quarter-wave plate and polarizer (see Fig 1). The spatial distributions of the values of Stokes vector parameters *S*_*i*=1;2;3;4_(*m*×*n*) were determined based on the following standard measuring procedure [4,7,18]:

- the polarization illuminator formed the series of linear (0^0^; 45^0^; 90^0^) and right- (⊗) circularly polarized illuminating laser beams;
- for each laser beam, the transmission axis of the polarizer was rotated by angles Ω = 0^0^;90^0^;45^0^;135^0^;
- for each value of Ω, the spatial distribution of the intensity of linearly polarized light $U_{0;90;45;135}^{0;45;90;⊗}\left(m\times n\right)$ was measured;
- the quarter-wave plate was placed in front of the polarizer; the fast axis of the quarter-wave plate was rotated by the angles Θ = 45^0^ and Θ = −45^0^ from the polarization transmission axis;
- spatial intensity distributions $U_{⊗;⊕}^{0;45;90;⊗}\left(m\times n\right)$ of the right (⊗) and left (⊕) circularly polarized light were measured by the camera for each angle Θ;
- spatial distributions of Stokes-vector parameters $S_{i=1;2;3;4}^{0;45;90;⊗}\left(m\times n\right)$ were calculated as follows:

$$\left(\begin{array}{l} S_{i=1}^{0;45;90;⊗}\\ S_{i=2}^{0;45;90;⊗}\\ S_{i=3}^{0;45;90;⊗}\\ S_{i=4}^{0;45;90;⊗} \end{array}\right)\left(m\times n\right)=\left(\begin{array}{l} U_{0}{}^{0;45;90;⊗}+U_{90}{}^{0;45;90;⊗};\\ U_{0}{}^{0;45;90;⊗}-U_{90}{}^{0;45;90;⊗};\\ U_{45}^{0;45;90;⊗}-U_{135}^{0;45;90;⊗};\\ U_{⊗}{}^{0;45;90;⊗}-U_{⊕}{}^{0;45;90;⊗} \end{array}\right)\left(m\times n\right)$$ (8)

Finally, the set of Mueller-matrix elements *M*_*ik*_ was calculated for each pixel of the camera:

$$\begin{array}{l} M_{11}=0.5(S_{1}^{0}+S_{1}^{90});\;M_{21}=0.5(S_{2}^{0}+S_{2}^{90});\;M_{31}=0.5(S_{3}^{0}+S_{3}^{90});\;M_{41}=0.5(S_{4}^{0}+S_{4}^{90});\\ M_{12}=0.5(S_{1}^{0}-S_{1}^{90});\;M_{22}=0.5(S_{2}^{0}-S_{2}^{90});\;M_{32}=0.5(S_{3}^{0}-S_{3}^{90});\;M_{42}=0.5(S_{4}^{0}-S_{4}^{90});\\ M_{13}=S_{1}^{45}-M_{11};\;M_{23}=S_{2}^{45}-M_{21};\;M_{33}=S_{3}^{45}-M_{31};\;M_{43}=S_{4}^{45}-M_{41};\\ M_{14}=S_{1}^{⊗}-M_{11};\;M_{24}=S_{2}^{⊗}-M_{21};\;M_{34}=S_{3}^{⊗}-M_{31};\;M_{44}=S_{4}^{⊗}-M_{41}. \end{array}.$$ (9)

The accuracy of the polarimetric measurement of the magnitude of the elements of the Mueller matrix is: *M*_*i* = 1−3;*j* = 1−3_ = 2%; *M*_*i* = 1−4;*j* =4_;*M*_*i* = 4;*j* = 1−4_ = 4% [18–19].

Using the (Eqs 8 and 9), the elements 〈{*m*_*ik*_}〉 of the differential matrix of the 1^st^ order (5) were determined for each pixel of the camera. Then, using the relations (6) and (7), the spatial distribution of the phase (Φ(*m*×*n*)) and amplitude (A(*m*×*n*)) anisotropy of fibrillar networks of biological tissues were found. Further in the text, polarization-phase spatial distributions are denoted as PT.

### Analysis of Mueller-matrix elements

Utilizing Eqs (6)–(9), obtained spatial distributions PT(*m*×*n*) were analyzed within the statistical approach [20]. By means of MATLAB software, we calculated the histograms N(Φ), N(A) (operator “hist”) and statistical moments of the 1^st^-4^th^ order (operator “mean”, “std”, “skewness”, “kurtosis”), which characterize the distributions **PT**(*m*×*n*):

$$Z_{1}=\frac{1}{K}\sum_{j=1}^{K}{\mathrm{PT}}_{j};Z_{2}=\sqrt{\frac{1}{K}\sum_{j=1}^{K}{\left(\mathrm{PT}-Z_{1}\right)}_{j}^{2}};Z_{3}=\frac{1}{Z_{2}{}^{3}}\frac{1}{K}\sum_{j=1}^{K}{\left(\mathrm{PT}-Z_{1}\right)}_{j}^{3};Z_{4}=\frac{1}{Z_{2}{}^{4}}\frac{1}{K}\sum_{j=1}^{K}{\left(\mathrm{PT}-Z_{1}\right)}_{j}^{4}$$ (10)

Here, *K* is the number of pixels on the CCD-camera. These parameters characterize the mean value(*Z*_1_), dispersion (*Z*_2_), skewness (*Z*_3_) and kurtosis or “peak sharpness” (*Z*_4_) of the histograms N(Φ) and N(A).

## Results and discussion

### Samples, preparation and statistical validation

We investigated samples of myocardium with necrotic conditions which are problematic to diagnose. The samples were provided by the Department of Pathology of the Oulu University Hospital from the collection used for teaching purposes; all the required consents and permissions for using the samples were acquired. The authors of the manuscript do not have any identifying information of the patients; none of the authors is a treating physician. The samples were taken as a part of routine care; they were not specially collected for the current study. The samples were assessed retrospectively.

The samples were divided into 2 groups: (i) myocardium of patients deceased as a result of the ischemic heart disease (IHD—group 1 "control") and acute coronary insufficiency (ACI—group 2 "diagnosed").

**Fig 2:**
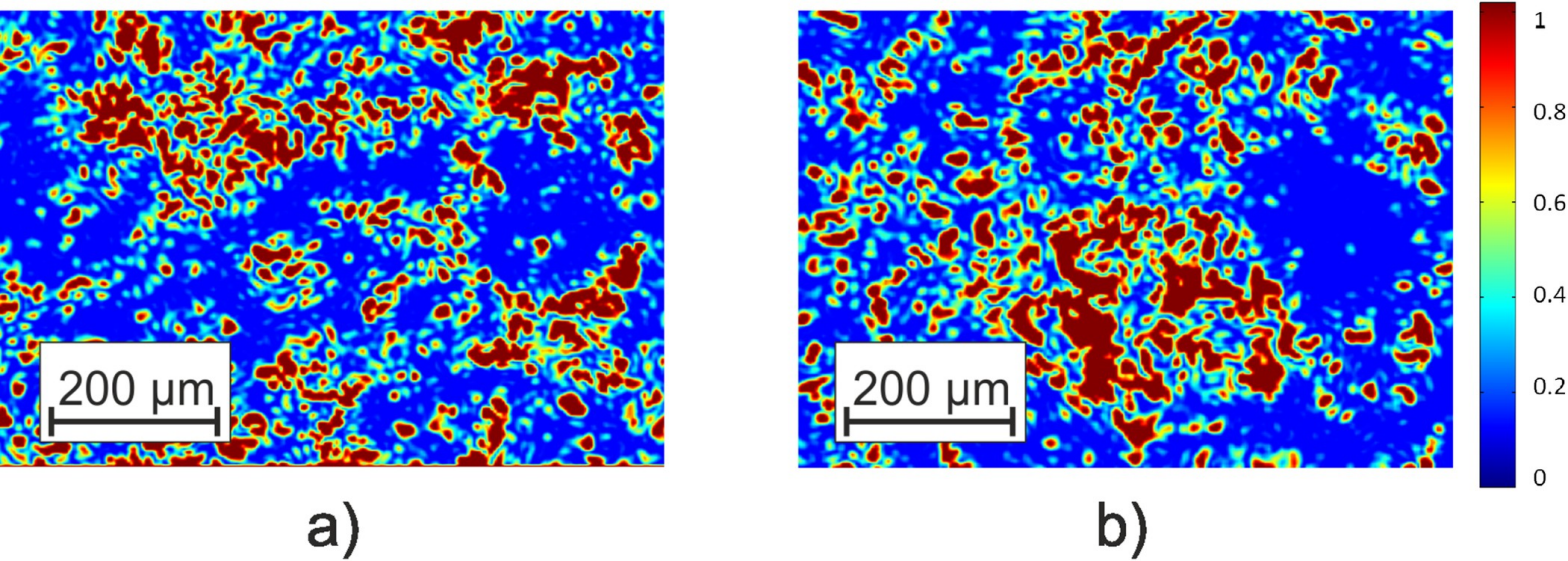
**Microscopic (4x) polarization-visualized images of the optically anisotropic component of the histological sections of the myocardium of control (a) and diagnosed (b) groups**.

The objects selected for the study combine the similarity in polycrystalline structure, namely, the presence of fibrillar networks (Δ*n*_0;90_;Δ*n*_45;135_;Δ*μ*_0;90_;Δ*μ*_45;135_), formed by optically active (Δ*n*_⊗;⊕_;Δ*μ*_⊗;⊕_) protein molecules of myosin. The comparative qualitative analysis of microscopic images revealed (see Fig 2): (i) the individual structure of polarization-visualized fibrillar networks of the histological sections of the myocardium; (ii) the absence of pronounced differences between the polycrystalline structures of all tissue samples within the control and investigated groups.

The differentiation of necrotic conditions of the myocardium histological sections was performed by the biopsy of surgically removed samples, which is believed to be a gold standard method. A number of 51 samples for all groups was determined as reliable by means of the Statmate software for the 95% confidence interval (*p*<0.05).

The samples of all biological tissues were prepared utilizing a freezing microtome according to the standard methodology. Most commonly used histological slices of optically thick (*l* = 30*μm*÷40*μm*) biological tissues were selected for the study. For all samples the multiple scattering regime was implemented: all the samples partly depolarize laser radiation (attenuation coefficient *τ*>0.01≈0.05÷0.07). Thus, the traditional laser polarimetry approach is limited to an approximation in the description of non-depolarizing optically thin layers (*τ*<0.01) [18–20] therefore, further generalization of Mueller-matrix mapping based on Eqs (3) and (5)-(9) is required.

### Mueller-matrix mapping of biological tissues histological sections

**Fig 3:**
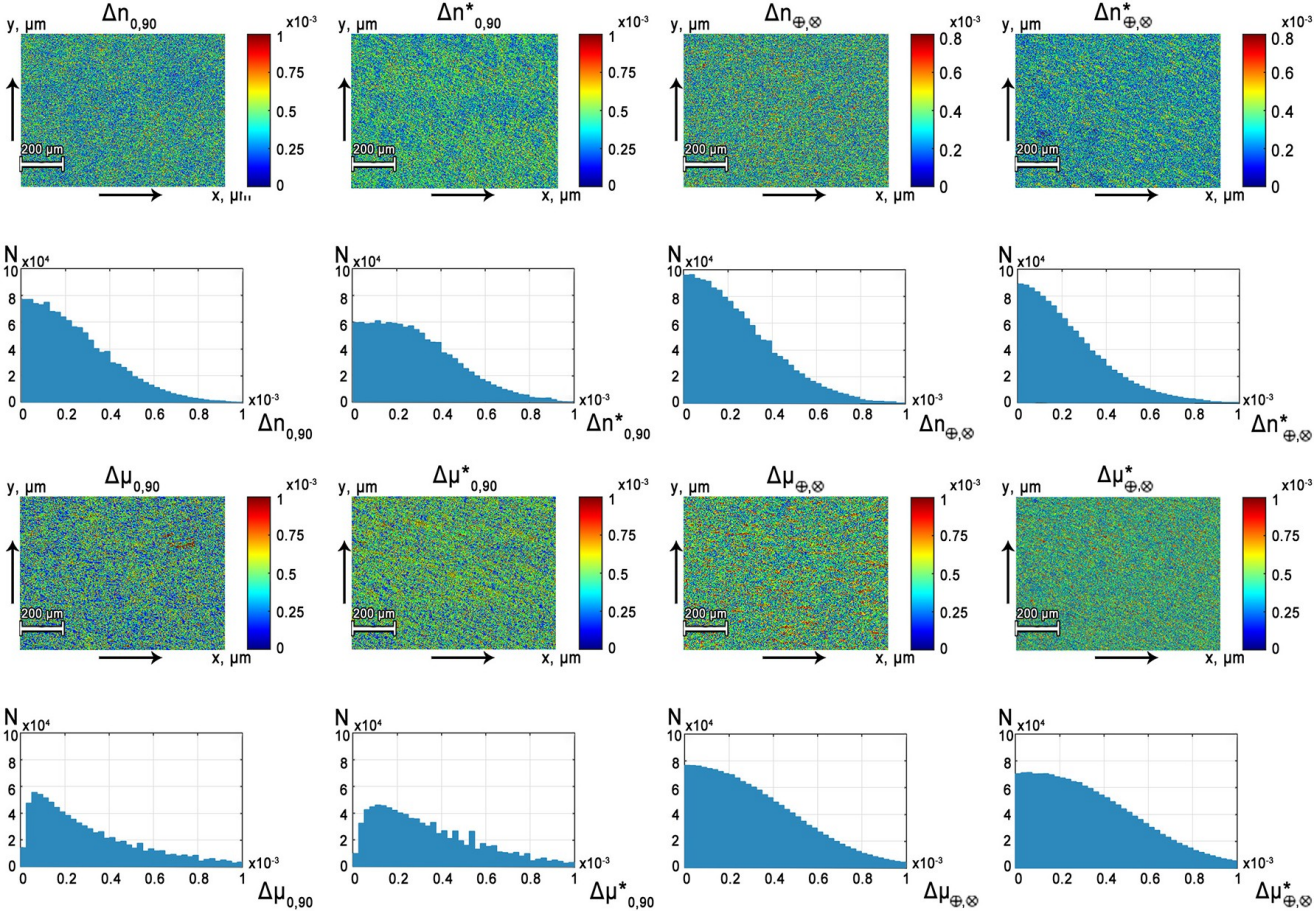
Polarization-phase spatial distribution PT(*m*×*n*) and histograms N(PT) of the distributions Φ(*m*×*n*) and A(*m*×*n*) of myocardium histological sections of deceased patients from the group 1 (Δ*n*_0;90_, Δ*n*_⊗;⊕_, Δ*μ*_0;90_, Δ*μ*_⊗;⊕_) and the group 2 ($\Delta n_{0;90}^{*}$, $\Delta n_{⊗;⊕}^{*}$, $\Delta \mu_{0;90}^{*}$, $\Delta \mu_{⊗;⊕}^{*}$).

Fig 3 present spatial distribution of polarization **PT**(*m*×*n*) and histograms N(**PT**) of the distribution of the phase and amplitude anisotropy of the myocardium.

The linear birefringence Δ*n*_0;90_ and dichroism Δ*μ*_0;90_ are more vivid compared to the circular phase Δ*n*_⊗;⊕_ and amplitude Δ*μ*_⊗;⊕_ anisotropy (see Fig 3). This is illustrated quantitatively by the lower values of the main extrema of N(Δ*n*_0;90_) = 0 and N(Δ*μ*_0;90_) = 0. Therefore, the larger average values ($\left\{ \begin{array}{l} \bar{\Delta }n_{0;90}>\bar{\Delta }n_{⊗;⊕};\\ \bar{\Delta }\mu_{0;90}>\bar{\Delta }\mu_{⊗;⊕} \end{array}\right.$) of histograms N(Δ*n*_0;90_) and N(Δ*μ*_0;90_) are formed. The comparison of the spatial distributions of polarization phase of the myocardium tissue samples has revealed the lower level of the linear birefringence and dichroism in the control group 1(IHD) - $\left\{ \begin{array}{l} \bar{\Delta }n_{0;90}\left(\mathrm{IHD}\right)<\bar{\Delta }n^{*}{}_{0;90}\left(\mathrm{ACI}\right);\\ \bar{\Delta }\mu_{0;90}\left(\mathrm{IHD}\right)<\bar{\Delta }\mu^{*}{}_{0;90}\left(\mathrm{ACI}\right) \end{array}\right.$ (see Fig 3), than in “pathology” group 2.

The obtained results are believed to be explained with accordance to the following physical considerations. Long process of IHD leads to the degenerative and dystrophic changes of the myocardium [28,29]. This is optically manifested by the decrease of the linear birefringence and dichroism due to the disorientation and reduction of the size of myosin fibrils [18–20]. Therefore, the distribution histograms N(Δ*n*_0;90_) and N(Δ*μ*_0;90_)(see Fig 3) obtained for histological slices of myocardium with IHD are characterized by the higher values (lower average *Z*_1_) of the main extrema of N(Δ*n*_0;90_) = 0 and N(Δ*μ*_0;90_ = 0). In addition, the half-width (dispersion *Z*_2_), as well as the skewness (*Z*_3_) and sharpness of the peak (kurtosis *Z*_4_) of such dependences are different.

In order to obtain quantitative estimates of the differentiation of the histological sections of the myocardium, the statistical analysis of the structure of polarization-phase images within both groups was performed.

### Statistical analysis

Here, we present the results of the statistical analysis of the data from two methods: the polarization microscopy (*I*_0;90_(*m*×*n*)) and the polarization-phase spatial distributions (Φ(*m*×*n*) and A(*m*×*n*)).

The differentiation between the groups of control (“1”) and investigated groups (“2”) was determined by using the following methodology [40–42]:

- within each set of values of statistical moments *Z*_*i* = 1;2;3;4_ (Eq (10)) we determined the average value ${\tilde{Z}}_{i=1;2;3;4}$ and standard deviation *σ*_*i* = 1;2;3;4_;
- differences between the statistical sets *Z*_*i* = 1;2;3;4_ were significant in the case when the average value ${\tilde{Z}}_{i=1;2;3;4}$ within the control group didn't "overlap" with the standard deviation *σ*_*i* = 1;2;3;4_ within the investigated group and vice versa;
- within both groups of biological tissues samples, the cutoff of 3*σ* (99.72% of all possible values of changes of *Z*_*i*_) was chosen for the distributions of values of the each statistical moment ${\tilde{Z}}_{i=1;2;3;4}$. Sequentially, we determined the number of "false negative" (*b*) and "false positive" (*d*) conclusions;
- for every statistical moment, the traditional for probative medicine operational characteristics^42^ were calculated: sensitivity ($Se=\frac{a}{a+b}100\%$), specificity ($Sp=\frac{c}{c+d}100\%$) and balanced accuracy ($Ac=\frac{Se+Sp}{2}$), where *a* and *b* are the number of correct and wrong diagnoses within group (“1”); *c* and *d* are the same within group (“2”) were determined.

#### Method of polarization microscopy

The results of the statistical and information analysis of the intensity distributions of the polarization-visualized images of the polycrystalline structure of biological tissues of all types are presented in Table 1.

**Table 1:** Parameters of the statistical analysis of polarization images of the polycrystalline structure of myocardium.

Tissue	Myocardium		
Parameters	Group 1	Group 2	*Ac*,%
*Z*_1_	0.32± 0.018	0.24± 0.016	68
*Z*_2_	0.25± 0.017	0.19± 0.011	70
*Z*_3_	0.37± 0.021	0.45± 0.026	66
*Z*_4_	0.45± 0.026	0.58± 0.034	67

The obtained results show insufficient level of accuracy of the polarization microscopy of myocardium necrotic changes. The value of balanced accuracy does not exceed 70% [29,30].

#### Method of polarization-phase imaging

The comparative analysis of the obtained data showed that the differences between the values of average ${\tilde{Z}}_{i=1;2;3;4}$ moments of all orders are statistically valid (Table 2).

**Table 2:** Parameters of the statistical analysis of myocardium polarization-phase images.

Parameters	Δ*n*_0;90_	Δ*n_0;90_	Δ*n*_⊗;⊕_	Δ*n_⊗;⊕_	Δ*μ*_0;90_	Δμ*_0;90_	Δ*μ*_⊗;⊕_	Δμ^*^_⊗;⊕_
	Group 1	Group 2	Group 1	Group 2	Group 1	Group 2	Group 1	Group 2
*Z*_1_×10^−3^	0.21± 0.00002	0.28± 0.000016	0.00025± 0.00016	0.003± 0.00016	0.007± 0.0004	0.005± 0.0003	0.0045± 0.00025	0.004± 0.00023
*Z*_2_×10^−3^	0.005± 0.00028	0.004± 0.0002	0.004± 0.0002	0.0035± 0.0002	0.008± 0.0006	0.006± 0.0004	0.006± 0.0004	0.005± 0.0003
*Z*_3_	0.39± 0.022	0.52± 0.033	0.48± 0.031	0.42± 0.024	0.71± 0.037	1.07± 0.0.69	0.28± 0.016	0.34± 0.019
*Z*_4_	0.51± 0.027	0.81± 0.044	0.67± 0.036	0.54± 0.029	0.44± 0.025	0.73± 0.038	0.17± 0.011	0.23± 0.015

However, there is an intergroup overlap for all histograms *N*(*Z*_*i*_). Moreover, the range of such an overlap is inversely proportional to the value of the difference between the averages ${\tilde{Z}}_{i=1;2;3;4}$. The moments *Z*_*i* = 3;4_(Δ*n*_0;90_) and *Z*_*i* = 3;4_(Δ*μ*_0;90_) appeared to be sensitive to the differentiation of linear birefringence and dichroism maps Δ*n*_0;90_(*m*×*n*);Δ*μ*_0;90_(*m*×*n*) of myocardium histological sections (highlighted in grey in Table 2). For the circular birefringence and dichroism, the polarization-phase images Δ*n*_⊗;⊕_(*m*×*n*);Δ*μ*_⊗;⊕_(*m*×*n*) of myocardium layers are less informative. The difference between the values of ${\tilde{Z}}_{i=1;2;3;4}$ in both groups of myocardium samples is not so vivid.

Table 3 presents the parameters of operational characteristics of the polarization-phase images of the optical anisotropy of histological sections of the myocardium biopsy with different necrotic changes.

**Table 3:** Operational characteristics of the method of the Mueller-matrix images of the optical anisotropy of the histological sections of the myocardium biopsy.

Parameters	*Z*_*i*_	Δ*n*_0;90_	Δ*n*_⊗;⊕_	Δ*μ*_0;90_	Δ*μ*_⊗;⊕_
*Ac*(*Z*_*i*_)	*Z*_1_	86%	62%	86%	61%
	*Z*_2_	88%	65%	68%	65%
	*Z*_3_	91%	78%	89%	72%
	*Z*_4_	95%	82%	94%	69%

The obtained results enable to state a rather high level of accuracy of the polarization-phase imaging. According to the criteria of probative medicine [39–41], the parameters *Ac*(*Z*_3_(Δ*n*_0;90_;Δ*μ*_0;90_))~90% correspond to the good quality, while *Ac*(*Z*_4_(Δ*n*_0;90_;Δ*μ*_0;90_))≻90% correspond to the high quality.

## Conclusions

The efficiency of the developed Mueller-matrix-based polarization imaging technique for the diagnosis of the necrotic changes of multiple scattering myocardium tissues has been introduced. The high-order statistical moments of distributions of the linear and circular birefringence, dichroism and their variations are utilized for the quantitative non-invasive assessment of the myocardium histological sections. We show that distributions of the phase and optical anisotropy formed by fibrillar networks of myocardium at different necrotic stages can be used as the quantitative diagnostic parameters. The differentiation criteria between the causes of death due to ACI and IHD were defined using the statistical analysis (statistical moments of the 1st– 4th order) of polarization-phase images of the polycrystalline structure of myocardium. The suggested quantitative approach is fast enough (the time of getting the result is *t*≤15 minutes) compared to the other techniques currently used in clinical practice. Thus, it has a strong potential for the application in histology for the differentiation of causes of necrotic changes in fibrillar tissues of various human organs. In the further studies, in order to implement this method into the routine laboratory practice in forensic medicine, a number of clinical tests are required.
